# Post-dengue transverse myelitis: a challenging case of neurological and therapeutic evidence

**DOI:** 10.1093/omcr/omag005

**Published:** 2026-02-24

**Authors:** Bianca Frigo Pires, Caroline Goulart Nunes de Souza, Eduardo Coviello Mendes de Campos, Talles Rajab, Mariana Ferreira de Albuquerque, João Victor Padovani do Carmo, Fernanda Ferraioli de Paula, Luiz Guilherme Peleckis, Bruno Tolentino Foroni, Clara Luisa Negraes Navarrete, Fernanda Goulart Nunes de Souza, Gustavo Henrique de Moura Vardasca, Natalia Seno Croccia, Raiza Marques Vieira Campos, Ruy Yoshiaki Okaji

**Affiliations:** Department of Neurosurgery, Faculdade de Medicina de Marília, Avenida Monte Carmelo, 800, Fragata District, Marília - State of São Paulo, 175190-30, Brazil; Department of Neurology, Faculty of Medicine of Marilia, Avenida Monte Carmelo, 800, Fragata District, Marília - State of São Paulo, 175190-30, Brazil; Department of Neurology, Faculty of Medicine of Marilia, Avenida Monte Carmelo, 800, Fragata District, Marília - State of São Paulo, 175190-30, Brazil; Department of Neurology, Faculty of Medicine of Marilia, Avenida Monte Carmelo, 800, Fragata District, Marília - State of São Paulo, 175190-30, Brazil; Department of Neurology, Faculty of Medicine of Marilia, Avenida Monte Carmelo, 800, Fragata District, Marília - State of São Paulo, 175190-30, Brazil; Department of Neurology, Faculty of Medicine of Marilia, Avenida Monte Carmelo, 800, Fragata District, Marília - State of São Paulo, 175190-30, Brazil; Department of Neurology, Faculty of Medicine of Marilia, Avenida Monte Carmelo, 800, Fragata District, Marília - State of São Paulo, 175190-30, Brazil; Department of Neurology, Faculty of Medicine of Marilia, Avenida Monte Carmelo, 800, Fragata District, Marília - State of São Paulo, 175190-30, Brazil; Department of Neurology, Faculty of Medicine of Marilia, Avenida Monte Carmelo, 800, Fragata District, Marília - State of São Paulo, 175190-30, Brazil; Department of Neurology, Faculty of Medicine of Marilia, Avenida Monte Carmelo, 800, Fragata District, Marília - State of São Paulo, 175190-30, Brazil; Department of Neurology, Universidade Federal de São Carlos, Rodovia Washington Luís, km 235, Monjolinho District, São Carlos – State of São Paulo, 13565-905, Brazil; Department of Neurology, Faculty of Medicine of Marilia, Avenida Monte Carmelo, 800, Fragata District, Marília - State of São Paulo, 175190-30, Brazil; Department of Neurology, Faculty of Medicine of Marilia, Avenida Monte Carmelo, 800, Fragata District, Marília - State of São Paulo, 175190-30, Brazil; Department of Neurosurgery, Universidade Federal do Amapá, Rodovia Juscelino Kubitschek, km 02, Jardim Marco Zero District, Macapá – State of Amapá, 68903-419, Brazil; Department of Neurosurgery, Faculdade de Medicina de Marília, Avenida Monte Carmelo, 800, Fragata District, Marília - State of São Paulo, 175190-30, Brazil

**Keywords:** infectious diseases and tropical medicine, neurology, critical care medicine

## Abstract

**Introduction:**

Dengue is an arbovirus with significant global prevalence that can lead to severe neurological complications, such as longitudinally extensive transverse myelitis (LEMT).

**Case report:**

We describe a 36-year-old man who developed sudden paraparesis and urinary retention approximately ten days after dengue infection. MRI revealed longitudinal hyperintense lesions in the cervical and thoracic spinal cord. Cerebrospinal fluid analysis revealed an inflammatory pattern and dengue serology was positive. The patient was treated with intravenous methylprednisolone and rehabilitation, leading to gradual neurological improvement and functional recovery.

**Discussion:**

Post-dengue transverse myelitis may result from parainfectious or post-infectious autoimmune mechanisms, often accompanied by demyelination and spinal cord inflammation. The low rate of DENV IgM in the CSF reinforces the immune-mediated hypothesis. Prognosis varies depending on the early intervention and the extent of spinal injury. Underreporting remains a challenge, and future studies should explore biomarkers and develop standardized management protocols.

## Introduction

Dengue is an arbovirus common in tropical and subtropical regions, with rising global incidence [1, 2]. Typical symptoms include fever, nausea, vomiting and abdominal or retro-orbital pain. Neurological complications may occur, including the rare and potentially fatal longitudinally extensive transverse myelitis (LETM) [3]. We present an important clinical case of LEMT in a young man diagnosed with dengue. The novelty in this case is that the early recognition and treatment—even only with steroids—resulted in rapid full recovery without permanent sequelae, highlighting the importance of recognizing these rare but serious manifestations of dengue and altering the myelitis outcome.

## Case report

A 36-year-old man, with no comorbidities and a history of alcoholism presented with fever, retro-orbital pain, headache, myalgia and generalized petechiae. These Dengue-like symptoms lasted for five days. He opted for symptomatic treatment at home with progressive improvement in the condition. Ten days later, he developed a sudden loss of strength in the lower limbs, accompanied by paresthesia and falling to the ground, without head trauma. The next day, there was bladder affection, and a catheter was placed.

On admission, he was in good general condition, flushed, hydrated, located and oriented in time and space. Muscle strength was grade V in the upper limbs and grade IV in the plantar flexion of the feet, with grade III in abduction and adduction. The weakness was distal and symmetric. Reflexes were exalted with bilateral patellar hyperreflexia and upper limb hyperreflexia. The sensitivity test showed paresthesia—patient presented loss of touch and temperature. Additional tests revealed indifferent Hoffmann, negative Babinski reflex and nasal index without dysmetria.

**Figure 1:**
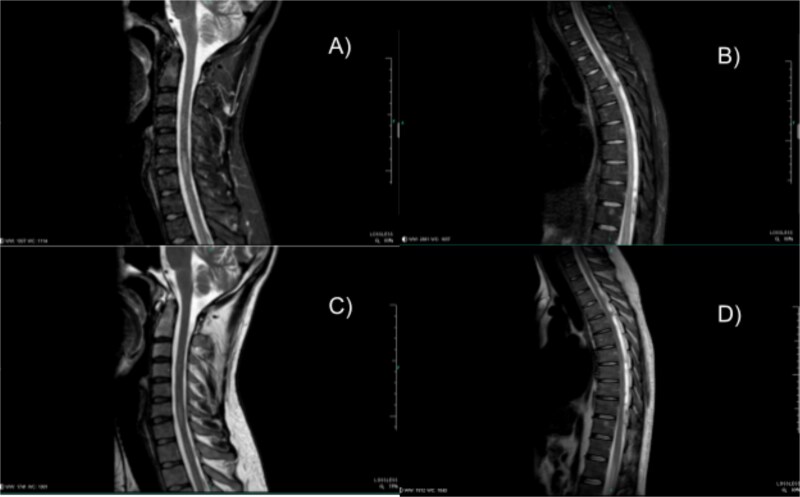
Spine MRI with hyperintense signal on T2 and STIR in the anterior aspect of the spinal cord at C6-C7, without gadolinium enhancement, and linear hyperintense signal in the thoracic spine, extending from T8-T10. (A) Sagittal STIR cervical. (B) Sagittal STIR thoracic. (C) Sagittal T2 cervical. (D) Sagittal T2 thoracic.

The investigation included analysis of the cerebrospinal fluid, which revealed increased cellularity (240 cells/mm^3^) with predominance of lymphocytes (89%), glucose level of 57 mg/dl and protein level of 52 mg/dl. Cranial and spinal tomography showed no signs of spinal cord compression, expansile lesions, extra-axial collections or pathological calcifications, and the brain parenchyma and ventricular system were normal. Magnetic resonance imaging (MRI) (Fig. 1) showed a hyperintense signal on T2 and STIR in the anterior aspect of the spinal cord at C6-C7, without gadolinium enhancement, and linear hyperintense signal in the thoracic spine, extending from T8-T10. As more than three spinal levels have been affected, it qualifies as LETM. Serology revealed reactive IgM and NS1 positivity for dengue. The patient tested negative for Hepatitis B and C, HIV, Neurocryptococcosis, Tuberculosis and Syphilis, excluding these differential diagnoses.

Given the clinical, laboratory and imaging findings, the diagnostic hypothesis of post-dengue transverse myelitis was formulated. The patient underwent clinical support with neurological surveillance and began pulse therapy with methylprednisolone 1 g intravenously for five days. Multidisciplinary monitoring started four days after the beginning of the symptoms and lasted until his discharge, thirteen days after, and it included motor physiotherapy four times a day and occupational therapy. During hospitalization, there was a progressive improvement in muscle strength in the lower limbs, with recovery of the ability to walk without assistance and reestablishment of bladder sphincter control. Such recovery excluded the need for escalation in the treatment course, despite the MRI findings suggestive of longitudinally extensive transverse myelitis. The patient was discharged in good clinical condition. During the outpatient follow-up three months later, he presented no greater deficit and recovered his muscle strength and tactile sensitivity. The neurological examination revealed grade V muscle strength in the upper limbs and grade IV in the lower limbs, symmetrical and preserved tactile sensitivity, and bilateral patellar hyperreflexia. The patient is in rehabilitation for mildly slowed gait but is improving clinically, with no further urinary retention.

## Discussion

Studies show post-dengue transverse myelitis can occur in both acute and postinfectious phases, appearing in days to weeks after fever resolution. Dengue-related transverse myelitis is a rare neurological event that can occur either from direct invasion of the central nervous system by the virus or from immune-mediated mechanisms, in which the immune and inflammatory response to the viral antigen triggers damage to the myelin of the spinal cord [2–4]. Studies indicate that post-dengue transverse myelitis ranges from progressive weakness in the lower limbs to severe autonomic dysfunction, including urinary and bowel retention. Additionally, a predisposition of the thoracic and cervical spinal cord involvement has been observed, which may directly affect the severity of symptoms [4, 5]. The radiological pattern of T2 hyperintensity and post-contrast enhancement reinforces the inflammatory hypothesis [6]. Magnetic resonance imaging remains the examination of choice for diagnostic confirmation, notably due to the presence of hyperintense lesions on T2-weighted images and post-contrast enhancement in extensive spinal cord regions [7, 8]. Such findings are consistent with active inflammation- that happened due to the autoimmune response—and reinforce the inflammatory hypothesis.

Therapy wise, corticosteroids remain the main approach, but there is a growing interest in adding immunomodulatory therapies, like intravenous immunoglobulins and plasmapheresis. Recent studies show that the early introduction of these therapies can reduce the progression of neurological conditions and improve functional recovery. Rituximab has also been reported in some cases of dengue associated with LEMT, particularly when an exacerbated autoimmune response is suspected [9].

The prognosis of patients with post-dengue LEMT varies. While some recover completely, a significant proportion remains with severe neurological sequelae, including persistent motor and autonomic dysfunctions. Favorable prognostic factors include early diagnosis, rapid treatment start, and a lesser extent of spinal cord injury on MRI. Recent reviews have shown that approximately 50% of patients experience significant functional recovery, while 30% remain with severe sequelae, such as paraplegia or autonomic dysfunction [10].

Epidemiological data suggest transverse myelitis in dengue is underreported, limiting accurate incidence estimates. Non-standard diagnostic criteria and limited access to advanced complementary tests are likely to contribute [10].

The underreporting of LEMT in dengue is a challenge, requiring greater epidemiological surveillance and its inclusion as a differential diagnosis in cases of neurological deficit after dengue infection. As we have experienced surveillance of neurological complications in dengue patients is essential for early diagnosis and less invasive interventions, resulting in better patient outcome.
